# Characterizing relaxin receptor expression and exploring relaxin’s effect on tissue remodeling/fibrosis in the human bladder

**DOI:** 10.1186/s12894-020-00607-4

**Published:** 2020-04-22

**Authors:** Edward C. Diaz, Mason Briggs, Yan Wen, Guobing Zhuang, Shannon L. Wallace, Amy D. Dobberfuhl, Chia-Sui Kao, Bertha C. Chen

**Affiliations:** 1 Department of Urology, Stanford University Medical Center, 300 Pasteur Drive, Grant S-287, Stanford, CA 94305 USA; 2 Present Address: Division of Pediatric Urology, Advocate Children’s Hospital, 8901 West Golf Road, Suite 301, Des Plaines, IL 60016 USA; 3 Department of Obstetrics and Gynecology, Stanford University Medical Center, 300 Pasteur Drive, Rm A370, MC 5317, Stanford, CA 94305 USA; 4 Department of Pathology, Stanford University Medical Center, 300 Pasteur Drive, Rm L235, Stanford, CA 94305 USA

**Keywords:** Relaxin, Fibrosis, Bladder, RXFP1, RXFP2

## Abstract

**Background:**

Relaxin is an endogenous protein that has been shown to have antifibrotic properties in various organ systems. There has been no characterization of relaxin’s role in the human bladder. Our objective was to characterize relaxin receptor expression in the human bladder and assess relaxin’s effect on tissue remodeling/fibrosis pathways in bladder smooth muscle cells.

**Methods:**

Relaxin family peptide receptor 1 (RXFP1) and RXFP2 expression was assessed using quantitative reverse transcriptase-PCR (qRT-PCR) and immunohistochemistry (IHC) on primary bladder tissue. Primary human smooth muscle bladder cells were cultured and stimulated with various concentrations of relaxin. Western blot, qRTPCR, ELISA, and zymogram assays were used to analyze fibrosis/tissue remodeling pathway proteins.

**Results:**

There was universal mRNA transcript detection and protein expression of relaxin receptors in primary bladder specimens. Immunohistochemistry demonstrated RXFP1 and RXFP2 localizing to both urothelial and smooth muscle cell layers of the bladder. 24 h of in vitro relaxin stimulation did not affect mRNA expression of selected proteins in human bladder smooth muscle cells. However, 48 h of in vitro relaxin stimulation resulted in upregulation of active (*p* = 0.004) and latent (*p* = 0.027) MMP-2 in cell lysate, and upregulation of active MMP-2 in supernatant (*p* = 0.04). There was a dose dependent relationship with increasing expression of MMP-2 with increasing relaxin concentration. Relaxin stimulation resulted in decreased levels of active and total TGF-β1 in supernatant and extracellular matrix (*p* < 0.005 with 100 ng/mL relaxin stimulation).

**Conclusions:**

In the human bladder, relaxin receptors are expressed at the dome and trigone and localize to the urothelium and smooth muscle cell layers. Stimulation of human bladder SMCs with relaxin in vitro affects expression of MMP-2 and TGF-β1.

## Background

Relaxin 2, commonly referred to as “relaxin”, is known in urology for its ability to facilitate closure of bladder exstrophy without osteotomy in the first 48 h of life. It binds to relaxin family peptide receptor 1 (RXFP1) and RXFP2 [1]. In addition to its ability to increase pelvic ligament and bone laxity in pregnancy, relaxin can inhibit fibrotic pathways [2] and modulate the extracellular matrix [3]. This antifibrotic effect has led to clinical trials looking at relaxin therapy for fibrotic medical conditions such as heart failure [4], scleroderma [5], and pulmonary fibrosis [6].

The poorly compliant bladder demonstrates a histologic phenotype of fibrosis. There is an evolution of initial hypertrophy and then loss of detrusor muscle [7], muscle replacement with collagen [8], and decreased elastin in the extracellular matrix [9]. Molecular pathways that contribute to this process are multifactorial [10]. Preliminary in-vivo data suggests a relationship between relaxin and bladder fibrosis. Using a radiated murine model, a group demonstrated that relaxin therapy reverses radiation-induced fibrosis and restores bladder function [11]. This evidence supports further investigation as to whether relaxin-related mechanisms also exist in the human bladder which, to our knowledge, has not been documented. The objective of this study is to characterize relaxin receptor expression in the human bladder and assess the in-vitro effect of relaxin on expression of proteins involved in tissue remodeling and fibrosis in human bladder smooth muscle cells.

## Methods

### Procurement of primary bladder specimens

IRB exemption and external approval from Donor Network West’s (federally designated organ procurement organization) Internal Research Council and Medical Advisory Board Research Subcommittee was obtained for collection of primary bladder tissue from brain dead cadaveric organ transplant donors. Brain dead donation was selected to eliminate warm ischemia time. Bladder tissue (Dome and trigone) was procured by a board certified urologist after removal of organs allocated for human transplant and immediately cryopreserved, placed in formalin, and processed into cell culture.

### Immunohistochemistry of primary tissue

Primary tissue was processed into paraffin blocks. 5 um sections were made and hematoxylin and eosin stain was performed. Slides were reviewed by a genitourinary pathologist.

### Immunofluorescence for RXFP1 and RXFP2 in primary tissue

5 um cryostat sections were fixed in 4% paraformaldehyde, permeabilized with 0.5% Triton X-100 in phosphate-buffered saline (PBS), and blocked in 1% bovine serum albumin. Primary antibodies: mouse anti-human cytokeratin (AE1/AE3) IgG (5μg/ml, Chemicon International), mouse anti-human desmin IgG (1:50, Sigma-Aldrich), rabbit anti-human RXFP1 IgG (10μg/ml, Abcam), rabbit anti-human RXFP2 IgG (10μg/ml, Lifespan Biosciences). Secondary antibody used: Alexa Fluor 488-conjugated goat anti-mouse IgG (1:300, Invitrogen), Alexa Fluor 594-conjugated donkey anti-rabbit IgG (1:200, Invitrogen).

### Quantitative reverse transcriptase-PCR (qRT-PCR)

RNA was extracted with RNA Stat-60 reagent (Tel-Test Inc.). Primers for target proteins were created using previously described protocols [12–16]. Samples were performed in duplicate. Brilliant SYBR Green qPCR Master Mix (Stratagene) was used. A 10 min hot start at 95 °C with forty cycles of 30 s of denaturation (94 °C), 1 min of annealing (60 °C), and extension at 72 °C for 30 s was used. Glyceraldehyde 3-phosphate dehydrogenase (GAPDH) was used as the endogenous reference. Human vaginal cuff tissue was used as a positive control for RXFP1 and RXFP2 expression.

### Bladder smooth muscle cell culture and Relaxin treatment

Primary human bladder smooth muscle cell (bSMC) line was obtained commercially from Lonza. Cells were grown in Dulbecco’s Modified Eagle Medium (DMEM) with 10% charcoal-stripped Fetal Bovine Serum (FBS) at 37 °C and 5% CO2 incubator. Cells (passage 4) were plated in triplicate. At 80–90% confluency, cells were then starved in serum-free DMEM with 0.2% lactalbumin enzymatic hydrolysate for 24 h, and then treated with various concentrations of human relaxin-2 (0–100 ng/mL, PeproTech) for 24 or 48 h as described previously by our group [17].

Supernatant was concentrated 100-fold using Vivaspin filtration tubes (Sartorius AG). To acquire proteins, cell layers were washed with (PBS) and lysed by 3 successive 10- min treatments with Complete-Mini Protease Inhibitor Cocktail (1 tab/10 mL, Sigma- Aldrich) in radioimmunoprecipitation assay (RIPA) buffer at 4 °C. Cell debris was removed by centrifugation.

Extracellular matrix (ECM) was prepared according to Pedrozo et al. [18] Cells were removed with RIPA buffer, the remaining cell-free, non-solubilized ECM was washed 3 times with PBS and plates dried overnight at room temperature. Isolated ECM samples were digested with plasmin (MP Biomedicals) in DMEM for 3 h at 37° to release TGFβ-118 [19]., Reaction was stopped by the addition of aprotinin (5 μg/mL, Sigma- Aldrich). Plasmin-digested ECM samples were collected and concentrated 200-fold with Vivaspin filtration tubes.

### ELISA measurement of active and latent TGF-β1

An ELISA kit (Promega Corp) was used. Nunc MaxiSorp 96-well plates were coated with TGF-β1 monoclonal antibody. Plates were washed and blocked and 100 μL of acid-treated and non-acid treated samples were then added to the wells in duplicate. After washing, anti-TGF-β1 polyclonal antibody was applied. Horseradish peroxidase HRP–conjugated antibody was used for detection. Absorbance at 450 nm was measured on a plate reader (Molecular Devices). Levels of both active and total TGF-β1 in the samples were normalized by protein concentration. Total protein concentration was determined using the Bradford method (Bio-Rad).

### Zymography of proteinases

Equal amounts of protein underwent sodium dodecyl sulfate polyacrylamide gel electrophoresis (SDS-PAGE) in 10% polyacrylamide containing 0.1% gelatin. Gels were soaked in 2.5% Triton X-100, incubated overnight at 37 °C in 0.05 mol/L Tris (pH 8.5), 5.0 mmol/L calcium chloride, and 0.02% sodium azide, then stained with 1% coomassie-blue R-250, 25% ethanol, and 15% formaldehyde. After staining, the enzyme activity appeared as clear bands against the blue-stained background. Activities of MMPs were identified by molecular weight. The area of lysis for each band detected was analyzed using Bio-Rad Quality One Software.

### Western blot

Protein from 24 h and 48 h supernatant and cell lysate were subjected to SDS-PAGE on 8% or 10% polyacrylamide gels. Gels were blotted onto nitrocellulose membranes. Blots were blocked and probed with mouse anti-TIMP-1 (1.0 μg/ml, EMD Bioscience) orTIMP-3 (3.0 μg/ml, EMD Bioscience). Secondary antibody was rabbit anti-mouse IgG (1:10,000, Amersham Pharmacia Biotech) conjugated to HRP. GAPDH was used as an endogenous reference. Membranes were re-blocked and reprobed with goat anti-GAPDH polyclonal IgG (1:500, Abcam) and HRP-conjugated mouse antigoat/ sheep monoclonal IgG (1:10,000, Abcam), for the primary and secondary antibodies respectively. Blots were developed by chemiluminescence.

### Statistical analysis

Statistical analysis for the dose–response studies was performed by using unpaired one-way ANOVA in IBM SPSS Statistics 21.0 software (IBM, Armonk, NY, USA). *P* value < 0.05 was considered statistically significant.

## Results

### Primary bladder procurement

**Fig. 1:**
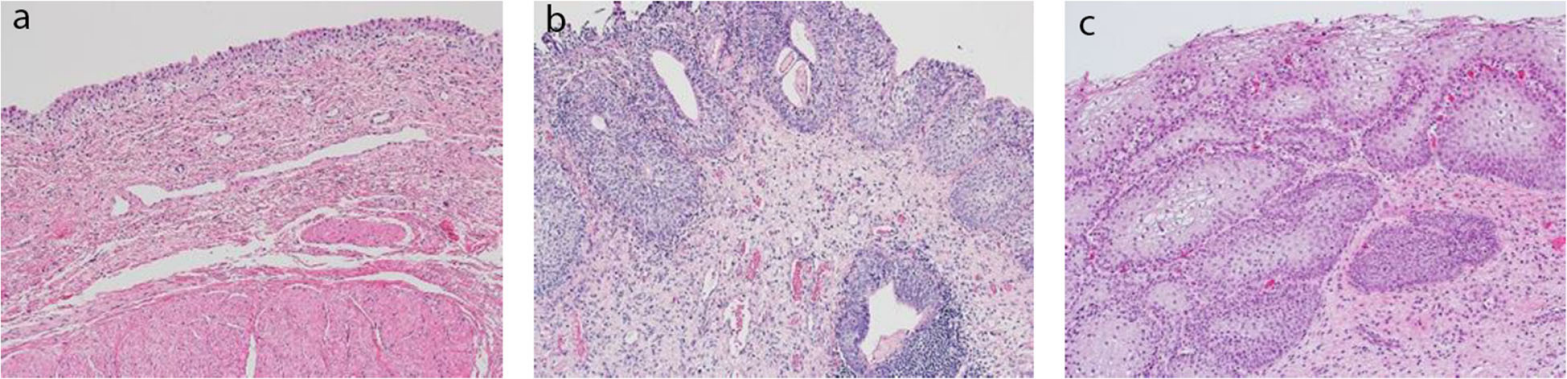
Hematoxylin and Eosin staining of donor bladders: **a** normal from donor 5 (10x), (**b**) cystitis cystica et glandularisfrom donor 4 (40x), and (**c**) non-keratinzing squamous metaplasia from donor 7 (40x)

Over a 6 month period, there were 26 offers for bladder tissue. Age range: 2–59, Gender: 17 male donors and 9 female. Patients with a history of a urologic condition, urologic surgery, malignancy, diabetes mellitus, elevated HGB A1c, were excluded. Eleven offers were accepted, but two were withdrawn due to hospital scheduling. A total of 9 bladders were procured. There were 5 male and 4 female donors with an age range of 2 to 55 years and mean age of 30.9 years and median age of 30 years. There was no warm ischemia time for donor tissue as organs were immediately flushed with ice cold UW solution and packed with ice after cross clamp. Cold ischemia time (Cross clamp to bladder tissue preservation) for the nine primary bladders was a mean of 68.6 min, with a median of 64 min. Pathology review for all donors confirmed benign urothelium, lamina propria and muscularis propria at the dome and trigone. Most bladders obtained were normal (Fig. 1a). Benign pathology was seen in donor 4 (Fig. 1b, cystitis cystica et glandularis), donor 7 (Fig. 1c, Squamous metaplasia), and donor 8 (mild chronic inflammation). A description of cadaveric donors is provided in Table 1.

**Table 1:**
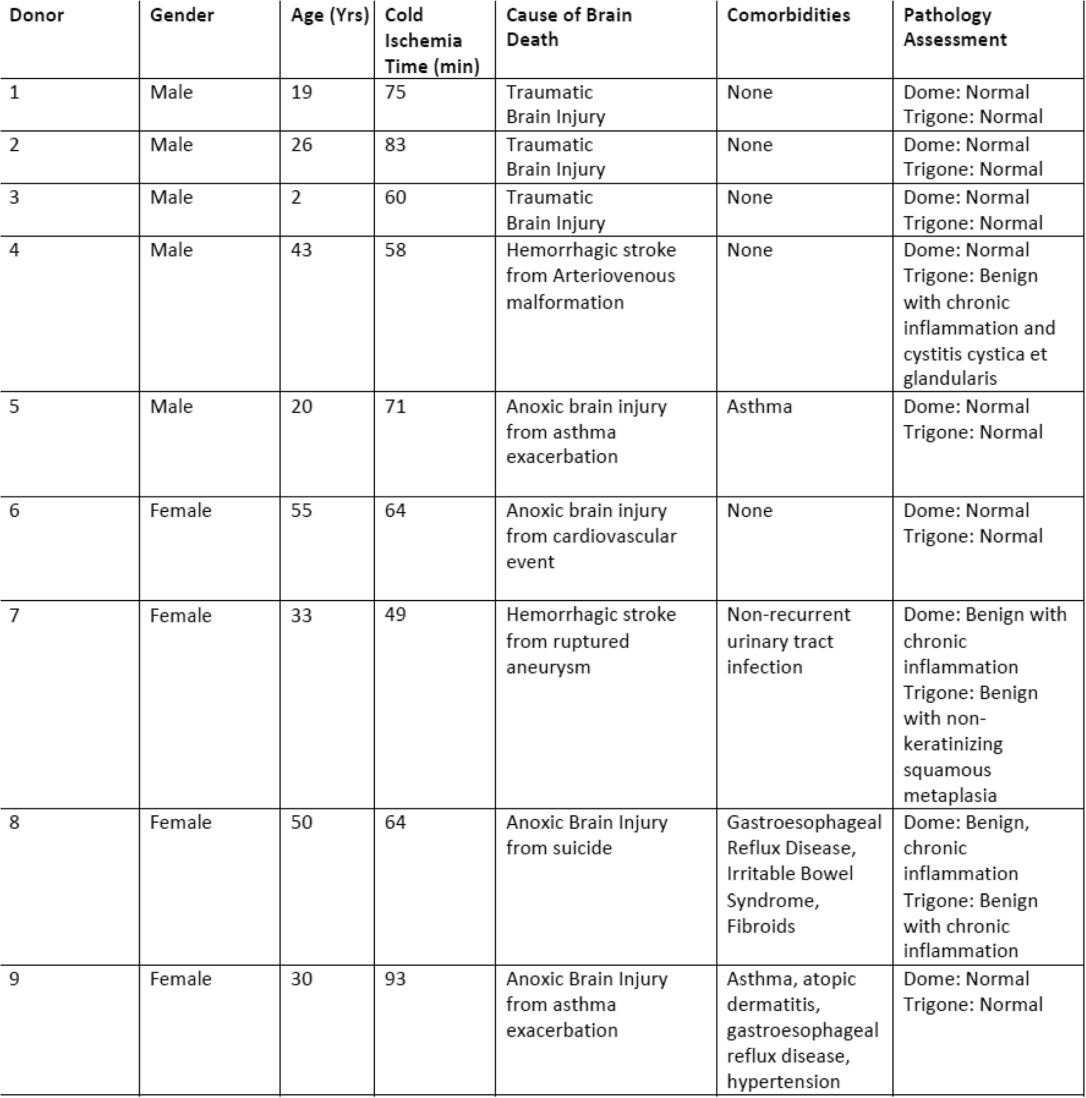
Cadaveric donor summary for primary bladder tissue

### Relaxin receptor expression

**Fig. 2:**
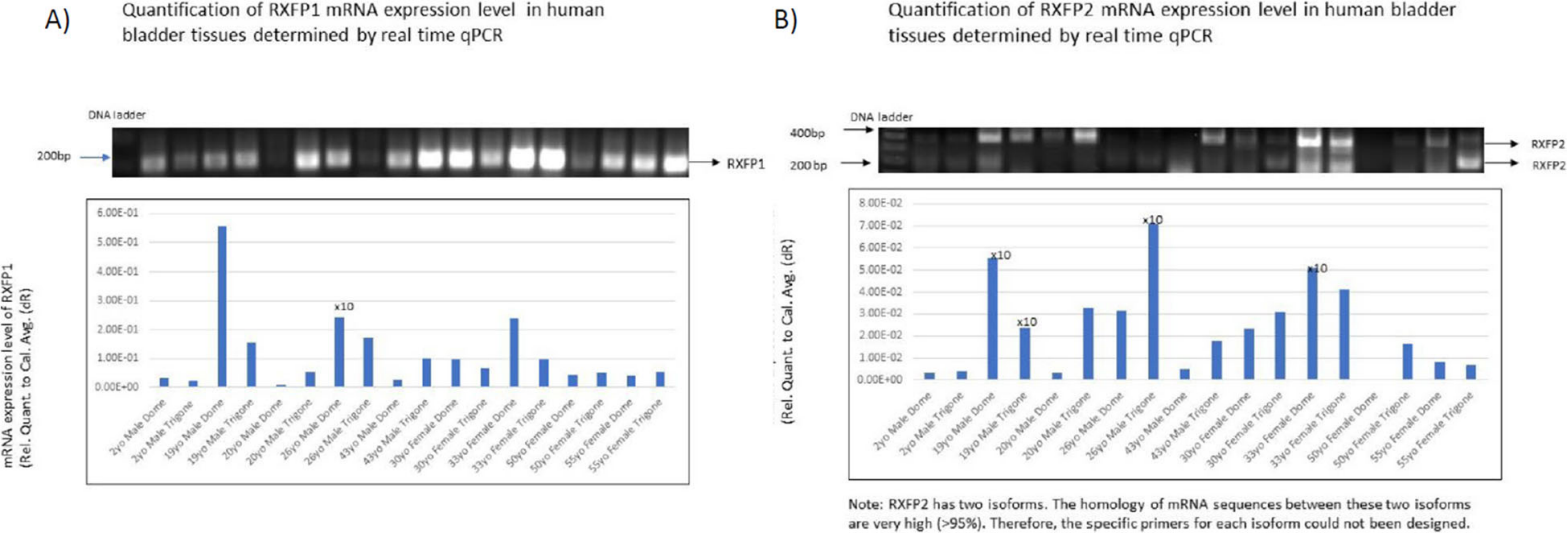
PCR results for Primary Human Bladders. **a** Primers amplified for RXFP1 demonstrate robust expression at the trigone, and dome. Quantification of expression was compared to PCR positive control of human vaginal epithelium. **b** Primers amplified for RXFP2 demonstrate amplification for all donors. Quantification of expression was compared to PCR positive control of human vaginal epithelium

*PCR:* qRT-PCR performed on dome and trigone for all 9 primary bladders (*n* = 18). All 18 samples demonstrated RXFP1 expression, and 17/18 samples demonstrated RXFP2 expression. The dome specimen for the 50 yo female donor did not have detectable RXFP2 transcript. The number of primary bladder specimens (*n* = 9) was too small to detect any significant differences between groups such as: male v. female, prepubertal v. postpubertal. The PCR data, however, confirms expression of both relaxin receptors at both trigone and dome. See Fig. 2.

#### Immunofluorescence

**Fig. 3:**
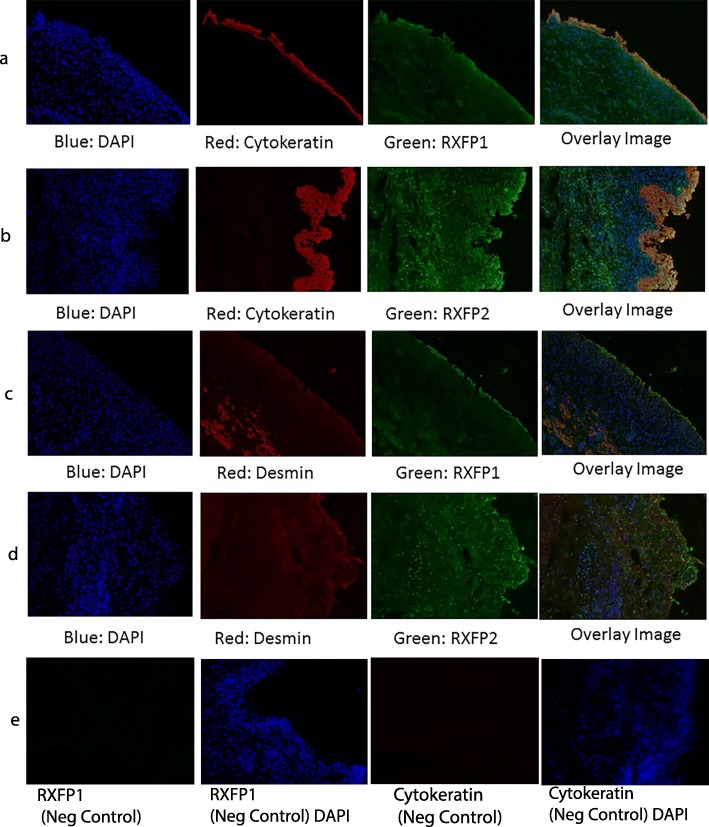
**a** Immunofluorescence for RXFP1 and Cytokeratin demonstrating urothelial localization (**b**) Immunofluorescence for RXFP2 and Cytokeratin demonstrating urothelial localization (**c**) Immunofluorescence for RXFP1 and Desmin demonstrating localization to lamina propria and muscularis propria (**d**) Immunofluorescence for RXFP2 and Desmin demonstrating localization to lamina propria and muscularis propria (**e**) RXFP1 negative control (primary antibody omitted) with DAPI and Cytokeratin negative control (primary antibody omitted)

Immune fluorescence demonstrated RXFP1 and RXFP2 expression within the urothelium, lamina propria and muscularis propria. This pattern of receptor distribution was consistent between male and female donors and RXFP1 and RXFP2 receptor distribution was similar between trigone and dome specimens. Figure 3a, and b demonstrates localization of relaxin receptors (RXFP1 and RXFP2, respectively) to urothelium and Fig. 3c, and d demonstrates localization of relaxin receptors (RXFP1 and RXFP2, respectively) to smooth muscle cell layers.

### Effect of relaxin on tissue remodeling and fibrosis pathways

#### Extracellular matrix proteins

**Fig. 4:**
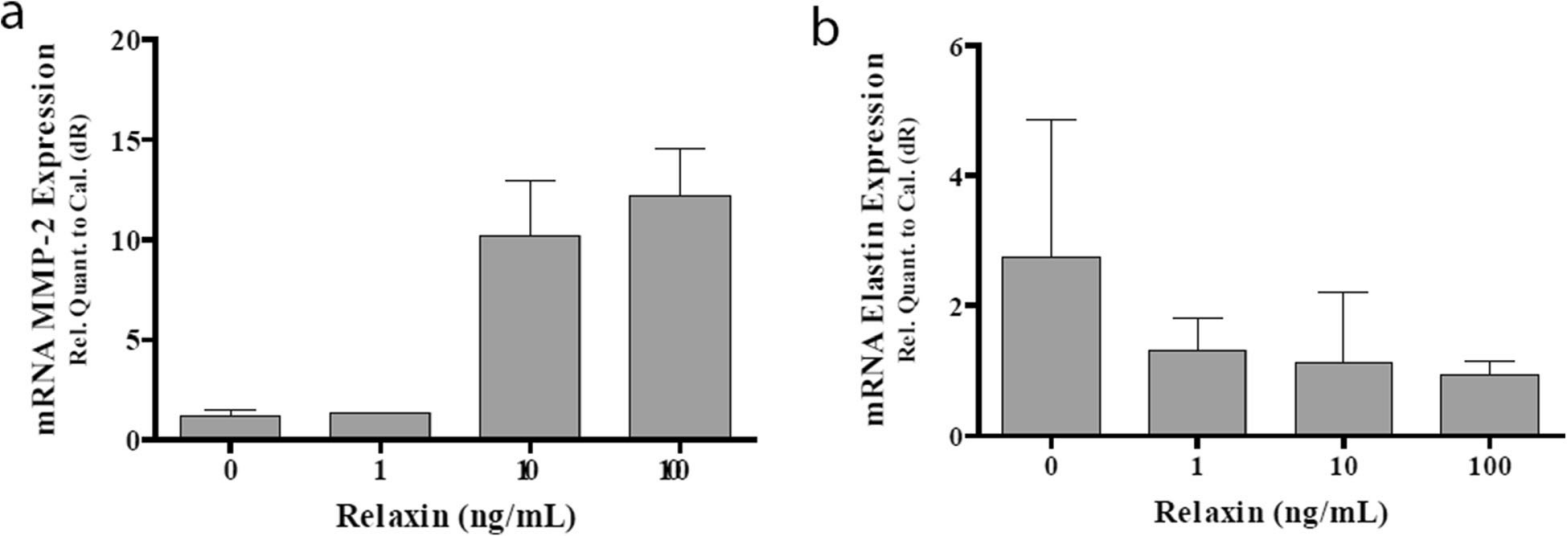
**a** mRNA MMP-2 Expression in 24 h Relaxin-2 treated human bladder smooth muscle cell lysate. **b** mRNA Elastin Expression in 24 h Relaxin-2 treated human bladder smooth muscle cell lysate

qPCR of cell lysate after 24 h of relaxin stimulation demonstrated no statistically significant change or discernible trend in expression for Collagen 1, Collagen 3, TGF-β1, TIMP-1, and TIMP-3. However, there did appear to be a dose dependent trend for MMP-2 and elastin that did not reach statistical significance. There was a dose dependent trend of upward expression for MMP-2 and a dose dependent downward trend in elastin expression. Refer to Fig. 4.

#### TGF-β1

**Fig. 5:**
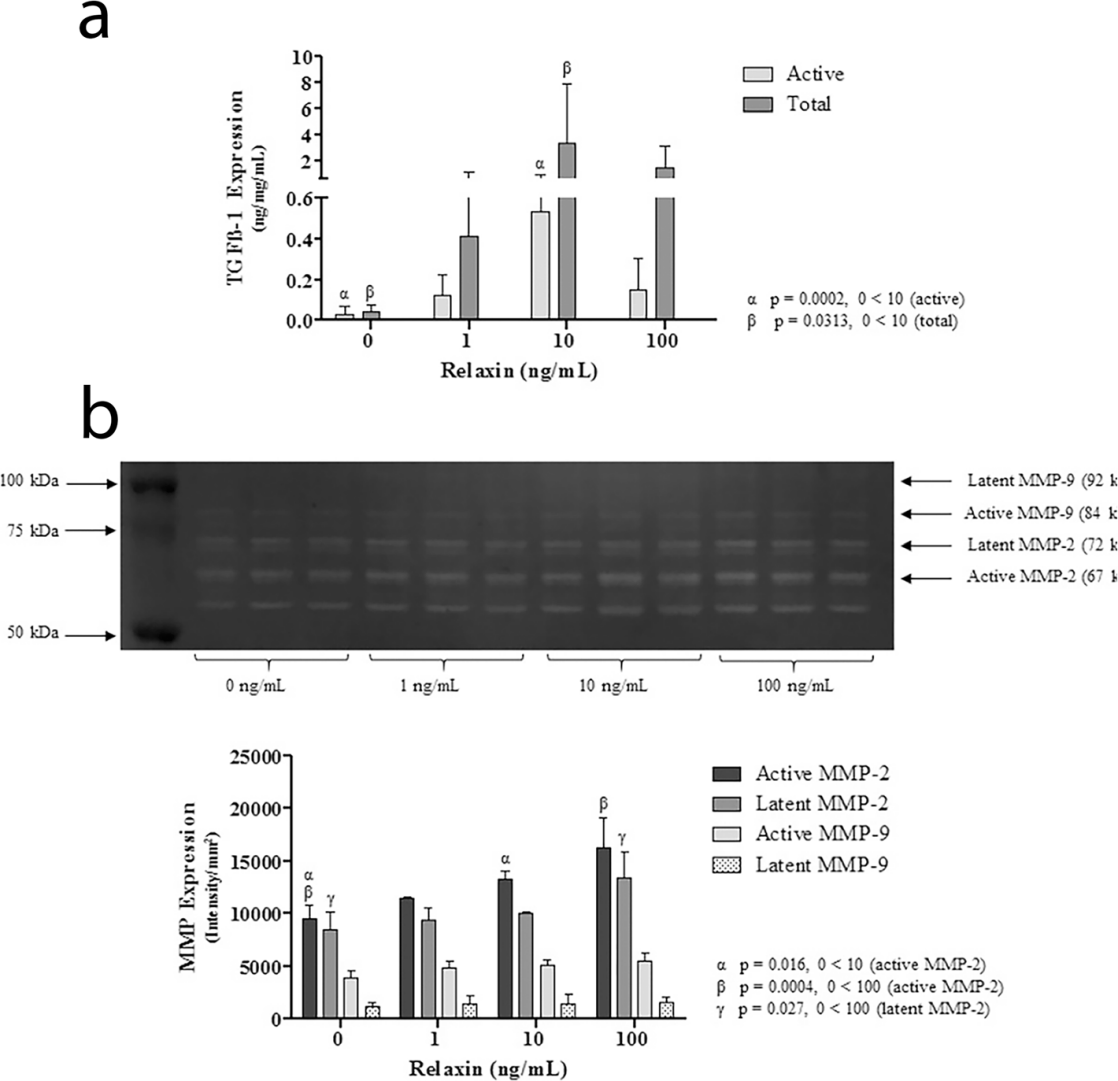
**a** TGF-beta 1 expression in cell lysate after 48 h of relaxin stimulation (**b**) MMP2 and MMP9 expression in cell lysate after 48 h of relaxin stimulation

In cell lysate, there was increase in active TGF-β1 and total TGF-β1 at all concentrations used for relaxin stimulation. Relaxin stimulation at a concentration of 10 ng/mL resulted in a 19 fold change (*p* < 0.005) in active TGF-β1 and an 82 fold change (*p* = 0.03) for total TGF-β1. See Fig. 5a. These results contrast with supernatant and extracellular matrix where there was a decrease in active and total TGF-β1. In supernatant, active TGF-β1 had a 0.12 fold change at 1 ng/mL and 0.27 fold change at 100 ng/mL (*p* < 0.005, < 0.005, respectively) and for total TGF-β1 there was a 0.10 fold change at 1 ng/mL, and 0.08 fold change at 100 ng/mL (*p* < 0.005, < 0.005, respectively). In extracellular matrix, active TGF-β1 had a 0.08 change at 1 ng/mL, 0.54 fold change at 10 ng/mL, 0.09 fold change at 100 ng/mL (*p* < 0.005, =0.01, < 0.005, respectively) and for total TGF-β1 there was a 0.9 fold change at 10 ng/mL (*p* = 0.01).

#### MMPs

At 24 h of stimulation there was no discernable trend and no statistically significant change in protein expression or activity for active MMP2 (*p* value range = 0.665 to 0.999). At 48 h there was no statistical change in active or latent MMP9 expression in cell lysate (*p* value range: 0.06 to 0.294 for active MMP9, *p* value range: 0.912 to 0938 for latent MMP9). In contrast, active and latent MMP2 expression increased in cell lysate and active MMP2 was increased in cell supernatant. See Fig. 5b. In supernatant active MMP2 had a 1.6 fold change at 10 ng/mL, and 1.7 fold change at 100 ng/mL (*p* = 0.08, =0.04, respectively). In cell lysate active MMP2 had a 1.4 fold change at 10 ng/mL, and 1.7 fold change at 100 ng/mL (*p* = 0.02, < 0.005, respectively). In cell lysate latent MMP2 had a 1.6 fold change at 100 ng/mL (*p* = 0.03, respectively). For cell lysate there was a dose dependent response with increased relaxin concentration resulting in increased MMP2 expression.

#### TIMPs

TIMP1 and TIMP3 expression was assessed in cell lysate at 48 h. There was no discernible trend for TIMP 3. In cell lysate there was an upward trend in TIMP1 expression. For TIMP-1 in cell lysate there was a 2.4 fold change at 1 ng/mL, 3.4 fold change at 10 ng/mL, and 2.1 fold change at 100 ng/mL (*p* = 0.56, =0.16, =0.73, respectively). Expression for TIMP-1 was also assessed in the supernatant at 48 h and did not demonstrate any trend or statistically significant change.

## Discussion

### Relaxin receptor expression in benign human bladder

Our results demonstrate RXFP1 and RXFP2 expression at the urothelium and muscularis propria within benign human bladder. Our in vitro data also provides preliminary evidence that relaxin signaling in bladder smooth muscle cells affects protein pathways involved in tissue remodeling and fibrosis. Further work is necessary to understand the role of relaxin signaling within urothelial cells. Urothelial signaling is an important component to overall bladder physiology and homeostasis. Urothelial pathways are regulated through mechanical stretch, and/or by molecules within the cellular and extracellular milieu, urine or blood. Molecular pathways that have been characterized for urothelial signaling include purinergic, muscarinic, nitric oxide, prostaglandin and nerve growth factor. These pathways affect cellular and neurovascular signaling within the lamina propria which in turn provides communication with the muscularis propria [20]. Given relaxin receptors ubiquity (dome and trigone and being present in all benign donor tissue), we suspect it plays a role in homeostatic bladder function. In addition to directly influencing fibrotic pathways, changes in urothelial relaxin signaling may impact urothelial homeostatic pathways and ultimately contribute to a pathologic phenotype, such as fibrosis. Researchers have suggested that changes in urothelial cell proliferation, permeability of the urothelium, and recruitment of immune cells mediate pathologic change [21]. Neurovascular signaling can also be impacted and can result in pathologic muscle activity.

The significance of two relaxin receptors in the bladder remains to be elucidated. RXFP1 and RXFP2 have similar protein structure and regulate adenylate cyclase and cAMP [1]. There is evidence in an animal model looking at knee laxity that RXFP receptor expression can be influenced by hormones such as testosterone and estrogen [22]. Hormonal regulation may be one explanation for two relaxin receptors being present in the bladder. INSL3 (primary protein substrate for RXFP2 [23]) signaling may also explain the presence of two relaxin receptors in the bladder. INSL3 is found in both men and women and it is consistently detectable in men and varies by pubertal and menopausal status in women. Its role has been mostly characterized in testicular descent and germ cell maturation. However, it is now being studied in various organ systems and has been found to affect bone metabolism and is elevated in conditions such as polycystic ovarian syndrome [24]. Characterization of relaxin receptor function is ongoing, and within the human bladder it will be essential to identify overlap, synergy or antagonism of RXFP1 and RXFP2 signaling. Researchers have developed fluorochrome and radioisotope tagged relaxin 2 and INSL3 and small molecules to selectively target individual receptors [25]. These molecules will be useful tools for the future.

Characterization of relaxin receptor expression in normal human bladder also provides important information on the appropriate animal model for future studies. Ideally the chosen animal model would have similar relaxin/relaxin receptor interaction and receptor distribution as the human bladder. In the mouse model of radiation cystitis previously mentioned [11], the histologic evaluation of relaxin receptor distribution in the mouse did not conform to our findings of urothelial expression in the human bladder. The mouse model, however, does illustrate both RFXP1 and RXFP2 in bladder smooth muscle cells similar to humans. Given differences in receptor distribution between human and mouse bladders, further exploration into the role of these receptors within the urothelium and smooth muscle layers can help determine whether a murine model best reflects human physiology. Various researchers have been assessing an appropriate animal model to study relaxin receptor signaling. One group found that macaque and pig models to have the most promise [26], but there are groups that have recently developed a transgenic mouse model to further preclinical study of relaxin [27].

Localization of the relaxin receptors to the urothelium, lamina propria, and muscularis propria also has significant translational implications. This suggests that intravesical instillation may be a potential route of administration for relaxin. In theory this would have less side effects compared to systemic administration. Systemic side effects were problematic in the phase 3 clinical trial looking at relaxin treatment of scleroderma. In that trial, patients were treated systemically with relaxin through a subcutaneous infusion pump for 24 weeks. Abrupt withdrawal of the relaxin treatment resulted in hypertension and decreased creatinine clearance and also resulted in menorrhagia [5].

### In vitro relaxin stimulation on primary human bladder smooth muscle cells

There are many molecular pathways involved in fibrosis. Frequently studied extracellular proteins, and signaling cascades were evaluated in our study. Our goal was to assess whether relaxin can affect tissue remodeling pathways in bladder smooth muscle cells derived from a person without bladder pathology.

After 24 h of stimulation there was no effect on transcriptional levels of studied proteins. This may have been due to lack of time to affect molecular change. Twenty four hours of stimulation with relaxin also did not result in changes to protein expression or activity. However, 48 h of stimulation did result in statistically significant findings. Both active and latent forms of MMP-2 were noted to be upregulated with relaxin stimulation. This upregulation was not demonstrated for MMP-9. In the literature, active MMPs digest surrounding matrix and create binding sites and molecules that can further promote or inhibit various biologic effects. Activation of mitogen activated protein kinase (MAPK)1 and MAPK3 by denatured extracellular matrix results in proliferation of bladder smooth muscle cells in vitro [28]. This proliferative effect on smooth cells as well as the ability of MMP2 to contribute to the breakdown of collagen can be viewed as an antifibrotic effect if one considers a fibrotic bladder to have decreased detrusor muscle and increased collagen deposition. The combination of increased MMP2 and decreased TGF-β1 has previously been described as anti-fibrotic in studies looking at heart failure [2, 3].

Results of relaxin stimulation at 48 h demonstrated increased levels of active and total TGF-β1 in cell lysate but decreased levels in supernatant and ECM. TGF-β1 is found within the cell, supernatant, and ECM. In the cell it is synthesized as an inactive/latent form that require endoproteolytic modification and ultimately binding of a latency TGF-β1 binding protein (LTBP) before it is secreted and deposited in the ECM. Within the ECM, latent TGF-β1 is stored for later activation. In order for TGFβ-1 to become active and bind to its receptor on the cell it needs to be released from its LTBP [29]. Understanding the complex processing and activation of TGF-β1 can help one conjecture the seen effects with relaxin stimulation. Increasing levels of TGF-β1 cell lysate can be related to decrease secretion and intracellular retention of the protein. In addition the decrease in supernatant and ECM can be explained by the presence of increased protein inhibitors of TGF-β1. Overall our findings of inhibition of the TGF-β1 pathway is similar to findings seen in heart failure. Within cardiac fibroblasts relaxin’s antifibrotic effect is believed to be due to inhibition of TGF-β1 Smad phosphorylation2 and inhibition of TGF-β1 Stat3 phosphorylation dependent autophagy [30].

## Conclusion

This data demonstrates the expression of relaxin receptors in the human bladder and that it localizes to both the urothelium, lamina propria, and muscularis propria and is expressed at both the trigone and dome of male and females of various age groups. Relaxin stimulation on normal human bladder smooth muscle cells affects expression of proteins involved in tissue remodeling and fibrosis. Further work is necessary to assess the role of relaxin and its receptors in pathologic bladder states.

## Data Availability

ED developed the idea of investigating relaxin in the human bladder, developed protocol for bladder tissue procurement and procured primary bladder tissue, and drafted the manuscript. BC helped design the study, served as a faculty mentor to ED, and provided lab space and personnel. CK provided pathology review of primary bladder specimens and provided Fig. 1 of the manuscript. MB, and YW designed and performed assays described in the methods section with the assistance of GZ, AD and SW. MB and YW also compiled the data, analyzed the data, and created figures and drafted the Methods section for the manuscript. All authors reviewed the manuscript and provided critical review and editing.
